# Flavor pleasantness processing in the ventral emotion network

**DOI:** 10.1371/journal.pone.0170310

**Published:** 2017-02-16

**Authors:** Jelle R. Dalenberg, Liselore Weitkamp, Remco J. Renken, Luca Nanetti, Gert J. ter Horst

**Affiliations:** 1 Top Institute Food and Nutrition, Wageningen, The Netherlands; 2 Neuroimaging Center Groningen, University Medical Center Groningen, Groningen, The Netherlands; Karl-Franzens-Universitat Graz, AUSTRIA

## Abstract

The ventral emotion network–encompassing the amygdala, insula, ventral striatum, and ventral regions of the prefrontal cortex–has been associated with the identification of emotional significance of perceived external stimuli and the production of affective states. Functional magnetic resonance imaging (fMRI) studies investigating chemosensory stimuli have associated parts of this network with pleasantness coding. In the current study, we independently analyzed two datasets in which we measured brain responses to flavor stimuli in young adult men. In the first dataset, participants evaluated eight regular off the shelf drinking products while participants evaluated six less familiar oral nutritional supplements (ONS) in the second dataset. Participants provided pleasantness ratings 20 seconds after tasting. Using independent component analysis (ICA) and mixed effect models, we identified one brain network in the regular products dataset that was associated with flavor pleasantness. This network was very similar to the ventral emotion network. Although we identified an identical network in the ONS dataset using ICA, we found no linear relation between activation of any network and pleasantness scores within this dataset. Our results indicate that flavor pleasantness is processed in a network encompassing amygdala, ventral prefrontal, insular, striatal and parahippocampal regions for familiar drinking products. For more unfamiliar ONS products the association is not obvious, which could be related to the unfamiliarity of these products.

## Introduction

The perceived pleasantness of flavors differs widely between individuals. Despite this variety, expressing affective responses to a flavor stimulus is very similar across individuals. This is reflected in characteristic positive facial expressions (e.g. lip licking and tongue protrusions) and negative facial expressions (e.g. gaping or making a facial grimace) in response to pleasant and unpleasant food stimuli, respectively [1,2]. Thus, although personal preferences may differ, changes in perceived pleasantness seem to be processed similarly across individuals.

To investigate the neural correlates of pleasantness processing, fMRI studies investigating affective responses to food cues mainly focused on "hedonic hotspots" in the brain using univariate analyses. These studies give useful localistic information about areas that are associated with the generation of affective responses [3]. However, affective responses are more likely generated by a network of brain areas that are functionally connected thereby advocating a network-oriented approach to investigate affective responses to flavors [4].

Based on reviewing lesion, stimulation and functional imaging studies investigating emotion perception in animals and humans, Phillips et al. (2013) [5] theorized a ventral emotion network encompassing the amygdala, insula, ventral striatum, and ventral regions of the prefrontal cortex. These authors related this network to the "*identification of emotional significance of environmental stimuli and the production of affective states*". A growing body of neuroimaging studies associated (parts of) this network with pleasantness coding of chemosensory stimuli: tastes, flavors, and odors e.g. [6–15] indicating that the ventral emotion network might also process the pleasantness of chemosensory stimuli. Interestingly, however, only a selected number of these areas is found to directly correlate with affective behavioral ratings of orally presented stimuli on a single study level (e.g. [7,16]). Instead, stimuli that are perceived as pleasant are contrasted against stimuli that are perceived as unpleasant in most studies. These studies still elucidate a small number of areas associated with pleasantness (e.g. [6,15,17,18]). Furthermore, most of the fMRI studies involving orally presented stimuli (including our own) required lenient statistical thresholds (e.g. no family wise error (FWE) corrections or small volume corrections) to elucidate involvement of these brain areas with affective processing. For example, in a previous study investigating the neural correlates of subjective flavor preferences, we found no differences between likers and dislikers of grapefruit juice using a FWE correction [15]. Although it seems obvious that taste and flavor processing requires some form of emotion processing to determine the emotional significance (e.g. the valence) of the stimulus, it remains unclear whether the ventral emotion network indeed processes flavor pleasantness.

In the current study we were specifically interested in affective processing of flavors during tasting. The perception of a flavor requires an integrative process involving multiple stimulus modalities such as gustation, olfaction, somatosensation, but also vision and audition [19]. Retronasal olfaction (i.e. sensing aroma volatiles originating from the oral cavity) is a very important component in this process [19]. Exhaling trough the nose induces retronasal olfaction. However, in the MR scanner, retronasal olfaction is mainly induced by swallowing[20]. As swallowing induces head and tongue movements, fMRI measurement of flavor perception is associated with substantial signal artifacts in the MR scanner.

In the current study, we analyzed two data sets in which affective brain responses to flavor stimuli were measured in young adult men. These men tasted regular off-the-shelf drinking products and Oral Nutritional Supplements (ONS) in the first and second data set, respectively. The different types of products were used to evaluate generalizability over different product categories. We included two datasets in order to investigate whether results acquired from one data set can be replicated in a second dataset. To shift the focus from individual hotspots to a network-based approach, overcome measurement sensitivity problems, and at the same time deal with movement artifacts induced by swallowing, we applied ICA at different stages during our analysis. This data-driven approach allowed us to discard signal artifacts and to divide the functional brain data during flavor processing into spatially independent functional brain networks. Subsequently, we related these functional brain networks to perceived pleasantness scores using linear mixed effect models and investigated whether the ventral emotion network is indeed associated with flavor pleasantness processing.

## Materials and methods

### General procedure

In the current study, we used fMRI data from a larger flavor experiment, which was divided into a screening session, an fMRI session, six daily repeated-exposure sessions, and a second fMRI session. In the screening session, inclusion and exclusion criteria were checked, and participants were familiarized with the experimental procedure. For the current analysis, we only used data from the first fMRI session because we intended to find neurobiological markers for taste pleasantness concurrent with tasting. Future studies are planned to focus on liking after repeated exposure. Participants were instructed not to eat or drink during a two-hour period prior to the fMRI session, which was scheduled between 8:00 and 12:00 am or between 4:00 and 7:00 pm. Results from the remainder of the study have been reported elsewhere (see e.g., [21]**)**

### Participants

A total of 45 male Caucasian university students were recruited for the experiment. Participation was on the basis of written informed consent and the medical ethical committee at the University Medical Center Groningen specifically approved this study. Participants were randomly assigned to two experimental groups. The first group (n = 23, mean age = 23.43, SD = 2.33, range: 21–28) was recruited to taste commercially available drinks (referred to as regular products) during a morning session. The second group (n = 22, mean age = 24.67, SD = 3.37, range: 21–33) was recruited to taste ONS products in the afternoon. These two groups were independent (i.e. no participant participated in both groups). Participants were included when they had no self-reported history of taste, smell, neurological, or psychological disorders. Participants were right handed, non-smoker for at least three months, and had normal or corrected to normal vision with MR-compatible lenses. Participants who had any psychiatric disorder, a history of drug abuse, non-removable metal on their body or who used any form of medication possibly affecting taste perception (i.e. gastrointestinal complaints, dry mouth, nausea, and taste disturbance) were excluded from the study. Participants received a monetary compensation for their participation.

Because food intake as well as brain responses to food images vary across the menstrual cycle (see e.g. [22,23]), we anticipated that inclusion of female participants within the study would introduce extra undesired variation, negatively affecting the analysis. Therefore, we only included male participants.

### Taste stimuli and delivery

The drinks were divided into two groups. The first group of drinks consisted of eight products that were commercially available in Dutch supermarkets at the time when the experiment was conducted. The drinks can be subdivided in two groups: four water-based drinks (flavors: apple-blueberry 27 Kcal / 100 ml, apple-peach 28 Kcal / 100 ml, orange-tangerine 27 Kcal / 100 ml and pineapple-mango 28 Kcal / 100 ml) and four yogurt drinks (flavors: raspberry 33 Kcal / 100 ml, coconut 32 Kcal / 100 ml, lemon 33 Kcal / 100 ml and orange-cinnamon 30 Kcal / 100 ml). The second group contained six ONS products. All six ONS products were milk based (flavors: apricot, vanilla and neutral, peach-ginger, cappuccino and orange-lemon, 160 Kcal / 100 ml). All liquids were administered at room temperature using a custom-made gustometer. This apparatus contained separate sterile syringes for each liquid, which were connected to a central mouthpiece by separate tubing for each liquid. Stimuli were administered manually. Timely stimulus administration was guaranteed by auditory countdown. See [24], for more details on the gustometer.

### Experimental design

**Fig 1:**
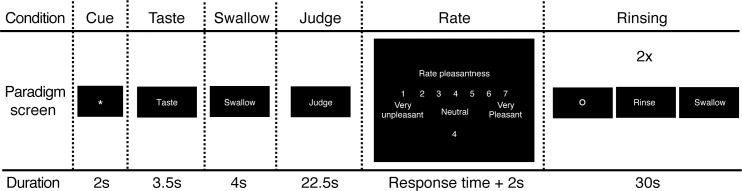
The trial structure of the fMRI taste paradigm. Every taste stimulus was delivered 6 times distributed over the entire paradigm. Every trial started with a Cue, followed by the Taste. The participant was subsequently instructed to Swallow, Judge and provide a pleasantness rating (Rate) for the stimulus. Every trial ended with two rinsing procedures.

The experimental design is similar to the experiment described in detail previously (see [24]) with several adjustments. A schematic overview of the fRMI paradigm is given in Fig 1. Participants engaged in a tasting task containing 48 or 36 trials for the regular products and ONS group, respectively. During the course of the experiment, participants received visual cues and instructions in Dutch via a paradigm constructed in E-prime (Psychology Software Tools Inc., Pittsburgh). Every flavor stimulus was delivered 6 times balanced over all imaging runs and counterbalanced between participants. The paradigm was presented during four and three imaging runs, for the regular products and ONS group, respectively. Each imaging run lasted for approximately 15 minutes (depending on reaction times) and contained a series of 12 trials. During each trial, participants were warned for an upcoming taste delivery by an asterisk appearing centered on the screen (duration: 2s.). Subsequently, 2 ml of a taste stimulus was delivered in the mouth and participants were instructed to taste this stimulus with the cue "Taste" (in Dutch: "Proeven", duration: 3.5s.). After tasting, participants were instructed to swallow the solution, cued as "Swallow" (in Dutch: "Slikken", duration: 4s.), followed by a period in which they needed to passively "Judge" the taste (in Dutch: "Beoordelen", duration: 22.5s.). We chose this long period to assure that BOLD responses associated with rating and tasting had minimal overlap. Finally, a 7-point Likert scale appeared on the screen, ranging from "very unpleasant" to "very pleasant". Participants were instructed to express perceived pleasantness of the taste on the scale by using a button box held in their right hand. Every trial ended with a rinsing procedure, in which participants received a 2 ml bolus of a 5% artificial saliva solution (Saliva Orthana, TM) twice. The entire paradigm lasted for approximately 90 minutes, in which either 288 ml or 216 ml of liquid was consumed, for the regular products and ONS group, respectively. As baseline, we included four 15-second periods in each imaging run within both data sets, during which the participant was looking at a black screen with a red cross centered in the middle.

### Data acquisition

MRI scans were performed using a 3-Tesla MR scanner (Philips Intera, Best, the Netherlands) equipped with a 32-channel head coil. A T1-weighted 3D fast field echo (FFE) whole brain image was obtained in transverse orientation for anatomical reference. Acquisition parameters: field of view (FOV) 256 × 232 × 170 mm^3^ (rl, ap, fh); voxel size 1 mm isotropic; TR = 9 ms; TE = 3.5 ms; flip angle 8°; SENSE factors: 2.5, 1 (ap, fh); 170 slices, scan duration = 246.3s. Functional brain images were acquired in sagittal orientation using the Principles of Echo-Shifting with a Train of Observations (PRESTO) sequence. Acquisition parameters: FOV 153 × 230 × 230 mm^3^ (rl, ap, fh); voxel size 2.87 × 2.87 × 3 mm^3^; TR = 20 ms; TE = 30 ms; flip angle 7°; SENSE factors: 1.9, 1.9 (rl, ap); scan time per volume 1.532s. As the experiment was self-paced, the number of volumes per imaging run ranged between 580 and 600. The data sets are available at https://openfmri.org as ds000218 and ds000219 for the ONS data set and Regular Products data set, respectively.

### Data analysis

Data was analyzed in R (http://www.r-project.org, version 3.1.3, 2015-03-09), SPM12 (Wellcome Trust Centre for Neuroimaging, (http://www.fil.ion.ucl.ac.uk/spm), and the GIFT Toolbox v3.0a (http://mialab.mrn.org) running in Matlab 2012b (The MathWorks Inc., Natick, MA). Functional images were registered to the mean functional image (i.e. realignment), co-registered the T1 image and normalized to the MNI template. Finally, the images were smoothed with an 8 mm full-width at half-maximum (FWHM) Gaussian kernel and resliced to a voxel size of 2x2x2 mm.

Due to technical difficulties with the gustometer or scanner, data was missing for several trials. We removed participants missing more than 25% of their data (3 ONS group, 1 regular drinks group). Furthermore, 1 participant in the regular drinks group was removed due to a brain abnormality. Therefore, fMRI analysis was performed on data from 19 and 21 participants for the ONS and regular drinks groups, respectively.

#### Pleasantness scores analysis

To provide insight on differences in scoring behavior between both datasets, we analyzed pleasantness-scoring behavior using linear mixed models (LMMs). LMMs are provided in R by the lmer-function from package lme4 (version 1.1–5, http://cran.r-project.org/package=lme4) [25,26]. Subsequent statistical tests on the LMMs were performed using the Satterthwaite’s approximation for the degrees of freedom, provided in the package lmerTest (version 2.0–11, http://cran.r-project.org/package=lmerTest) [27]. We tested both the difference in mean pleasantness-score as well as the difference in pleasantness-scoring behavior as a function of time during the experiment between both data sets. Within the model, pleasantness scores were entered as dependent variable while dataset, trial number, and their interaction were entered as independent variables. Finally, the variable participant constituted a random variable.

#### Independent component analysis

Independent component analysis (ICA) is a technique that extracts signal sources from a mixture of signals. McKeown and Sejnowski (1998) [28] introduced ICA in the spatial domain for fMRI analysis. Spatial ICA (sICA) assumes that each voxel contains a mixture of source signals. By grouping all voxels that elicit co-occurring signals, the measured signal mixtures are separated into spatially independent voxel patterns, termed independent components (ICs). Each IC represents a functional brain network [28–30]. Since its introduction, spatial ICA has been used as a technique for extracting functional networks from fMRI data [29]. An additional advantage of spatial ICA is its ability to isolate signal sources originating from MRI artifacts. These artifactual signal sources such as head and tong movement, MR scanner noise and cardiac and respiratory pulsations show spatially independent characteristics. [28,29]. In the current study, we made use of both strengths of spatial ICA by applying it for artifact removal prior to first-level analysis and for task-specific functional network extraction at second-level [31].

#### ICA artifact removal

To deal with artifacts associated with swallowing-induced head motion, we applied an ICA artifact removal (ICA-AR) method before first-level analysis, analogously to implementations in [32,33]. This method allows separating artifact related spatial components from components associated with neural processing. First, we reduced data dimensionality using two principal component analysis (PCA) data reduction stages; on subject-level, data was reduced to 45 principal components (PCs), after which data from all subjects were concatenated in time and reduced from 45 to 30 PCs at group-level. Note that the optimal number of ICs is often determined by the minimum description length (MDL) algorithm while using the GIFT Toolbox [34]. As the total number of volumes over all imaging runs in our scanning paradigms approximately ranged from 1750 to 2350 volumes per participant, such an algorithm produces high component estimations (~90) producing components that reflect specific brain areas that poorly correlate with full brain white matter and CSF probability maps. Therefore, we chose to estimate 30 ICs, which approximates the number of components extracted by Kim et al (2008) [32].

**Fig 2:**
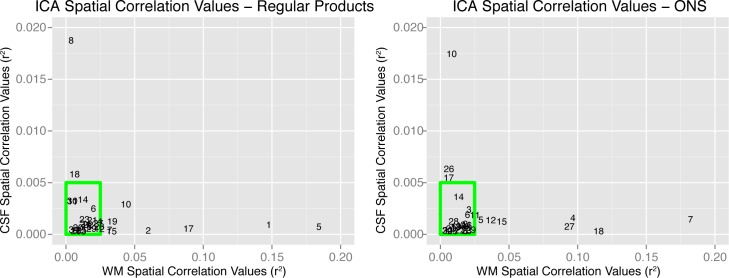
Removing artifact signals using spatial correlations. During the analysis, artifacts were removed using independent component analysis (ICA). To identify artifactual independent components (ICs), ICs were spatially correlated to prior white matter (WM) and cerebral spinal fluid (CSF) maps. The figure indicates spatial correlation values (Pearson r^2^) between ICs and CSF (vertical axes), and between ICs and WM (horizontal axes). Higher correlation values indicate higher spatial overlap between an IC and WM or CSF. Components within the green boxes were retained for cleaned fMRI signal reconstruction.

**Fig 3:**
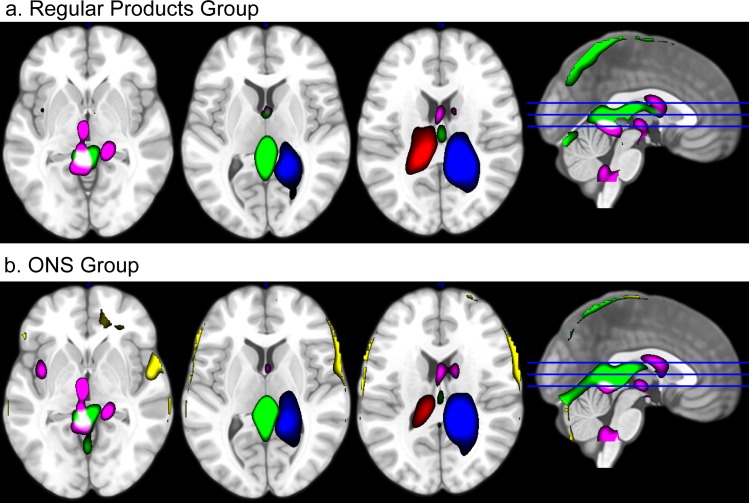
Spatial examples of removed artifacts. During the analysis, artifacts were removed using independent component analysis (ICA). The figure illustrates an overlay of 4 (a) and 5 (b) out of 10 spatial independent components (ICs) that were removed from the data for the regular products group and ONS group, respectively. These ICs represent various types of artifactual influences on fMRI signal, such as movement related artifacts (green, yellow) and artifacts originating from cerebral spinal fluid (pink, blue, red). For visual comprehension, only a subset of ICs is shown. Colors refer to specific ICs. Component maps are thresholded at z > 2.

Spatial ICs were estimated using the Infomax algorithm [35]. ICs were subsequently spatially correlated with prior probabilistic maps of white matter and cerebral spinal fluid (CSF) within the standardized MNI brain space, provided in SPM12. We thresholded spatial correlation values at r^2^ = 0.025 and r^2^ = 0.005 for white matter and CSF, respectively. These threshold values were based on visual inspection of the components. Components showing high correlations (r^2^) with these maps were considered artifacts and removed from the data. Fig 2 indicates the spatial correlation of all components with WM and CSF probability maps. For both data sets, we identified 10 components having considerable spatial overlap with WM and CSF. Two subsets of the removed components are given in Fig 3 showing an example of the various artifactual signals that have been removed from the data. The 20 retained components were used for "cleaned" fMRI signal reconstruction using the GIFT Toolbox. These data sets were used for first-level statistical analysis.

#### First-level statistical analysis

For the first-level statistical analysis, we constructed mass-univariate general linear regression models for each participant. The regressors included: 1) conditions 'Taste & Swallow', 'Judge', and 'Rate' for each taste trial separately, allowing subsequent modeling of separate trials on second level, 2) a global condition 'Rinse', and 3) the realignment parameters and their first derivatives as covariates [36]. The task-related regressors were convolved with the canonical hemodynamic response function (HRF) and a high-pass filter of 128 seconds was applied. Convolved regressors showed a maximum colinearity of (Pearson) r = 0.51.

Similar to the study reported in [24], we experienced technical difficulties with the PRESTO sequence. As a result, several PRESTO images were missing during the first fixation cross of multiple fMRI runs (ONS: 5 out of 57 fMRI runs; RP: 4 out of 78 fMRI runs; on average 0.054% per data set). To remove timing effects of missing volumes, we replaced these volumes with the first PRESTO volume of the imaging run to fill the gap in the time series, and included a separate regressor (of no interest) for each missing volume in the first-level statistical analysis such that it did not affect effect size estimates.

#### Second-level mass univariate analysis

On a group level, we carried out a regular mass univariate analysis in SPM. This analysis served two purposes. First, we contrasted the average brain response to flavor stimuli delivery versus baseline as a control step to ensure we successfully measured brain responses related to tasting a flavor. Resulting activation maps were thresholded at a global family-wise error (FWE) probability of P_(FWE)_ < 0.05. Second, although it was not our main aim to compare ONS versus regular products, we contrasted the average brain response to flavor stimuli in both data sets to indicate possible differences between the groups that might have influenced our second level ICA results. We did not find any results using a threshold of P_(FWE)_ < 0.05 on a full brain level and as we did not have a strong hypothesis about brain regions differentiating the participant groups prior to our analyses, we did not apply a ROI analysis nor a small volume correction. For completeness, we will report results on these group differences with a more lenient threshold of P_(uncorrected)_ < 0.001 but we will not draw profound conclusions based on these results.

#### Second-level ICA

In the current study, we aimed to extract functional networks associated with flavor pleasantness processing during flavor perception including tasting and swallowing. Therefore, we applied ICA for extracting task-specific functional networks on second-level (see Calhoun and Allen (2013) for a validation of second-level ICA). Compared to classical mass-univariate analysis of fMRI data, this approach provides several major advantages: first, it reduces the multiple-comparisons problem within mass-univariate analysis; second, by using blind source separation, the response dynamic to flavor pleasantness need not be exactly specified beforehand; and third, several studies suggest that spatial ICA is more sensitive in detecting task-related changes in fMRI signal than traditional mass univariate approaches [29,37].

To keep the analyses separate, we performed second-level ICA independently on both data sets. We used the 48 (regular products group) or 36 (ONS group) beta maps of the 'Taste & Swallow' (flavor) condition from the first-level statistical analysis as input data, we excluded the "Judge" period to exclude potential BOLD signal variability in response to differences in aftertaste from trial to trial. First, these beta maps were mean centered) in the spatial domain. Subsequently, the number of ICs was estimated using the MDL algorithm [34], which resulted in 15 and 13 ICs, for the regular products group and ONS group, respectively. For the regular products, group data was reduced to 23 PCs on an individual level and to 15 PCs on a group level, while for the ONS group, data was reduced to 20 PCs on individual level and subsequently to 13 PCs on group level. These differences arise due to differences in available data points (beta maps) for both data sets.

Note that ICA is commonly performed on fMRI runs. Results of these ICA's contain information in a spatial domain (functional brain networks) and a time domain (a time-course per network). However, on second (group) level, there is no "time domain" but a "flavor condition domain" representing a profile of IC loadings that indicate how strong each IC is represented in each condition (i.e. flavor trial) per participant. We will refer to these loading profiles as flavor condition profiles.

As we kept both datasets separate, between group differences may be more difficult to interpret. Therefore, we carried out an identical analysis performed on both groups together. Results of this ICA are found in S1 Fig.

#### Relating independent component maps to flavor pleasantness

In order to relate the resulting ICs of each data set to flavor pleasantness, we applied additional LMMs. For all constructed models, flavor condition profiles were entered as dependent variables, while flavor pleasantness scores, centered for each participant, constituted the independent variable. Finally, the participants and flavor quality constituted as random variables in the LMMs. These random variables corrected for possible systematic differences between participants and stimulus types [38]. As we tested the relation between flavor condition profiles and flavor pleasantness per IC (15 in the regular products data set and 13 in the ONS dataset), we applied an FDR correction on the resulting LMM P-values.

## Results

### Behavioral results

**Fig 4:**
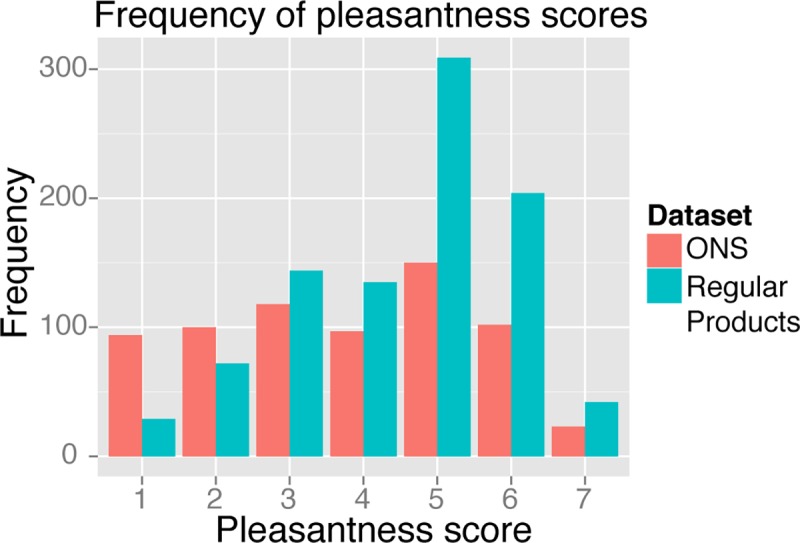
Flavor pleasantness scores. Histogram indicating the frequency of all pleasantness scores within the ONS (mean = 3.74, sd = 1.75) and regular products (mean = 4.5, sd = 1.45) data sets. Regular products received higher pleasantness scores (β = -0.75, T(37.58) = -3.61, P < 0.001).

To give an overview of the pleasantness scores, frequencies of scores are given in Fig 4. The LMM on the pleasantness data indicated that the drinks in the ONS dataset were perceived as less pleasant than in the regular products dataset (β = -0.75, T(37.58) = -3.61, P < 0.001). Furthermore, pleasantness scores significantly decreased per trial in the ONS dataset (β = -0.02, P < 0.001), whereas scores did not significantly change in the regular products dataset (β = -0.003, P = 0.40). The difference between these trial-effects in both datasets was significant (β = 0.02, P < 0.05). Data is available in S1 Dataset and S2 Dataset.

### Functional MRI results

#### Second-level mass univariate analysis

**Fig 5:**
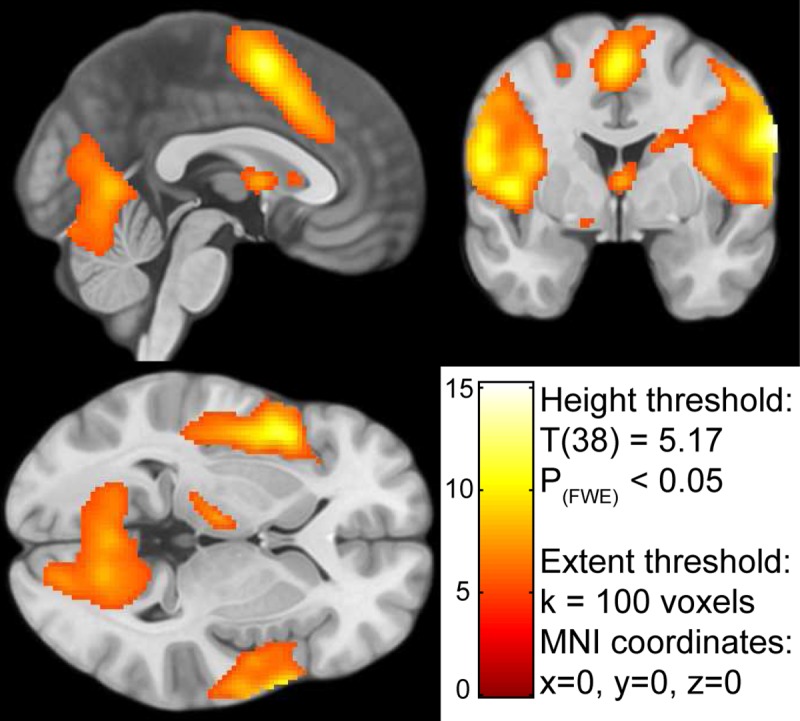
Result for the SPM mass univariate second level analysis. The result indicates the activated voxels in response to tasting a flavor during the fMRI scan. The color-coding of the intensity map is based on the T value generated by the contrast [Taste & Swallow—baseline]. The contrast map is available in the supplementary materials.

**Fig 6:**
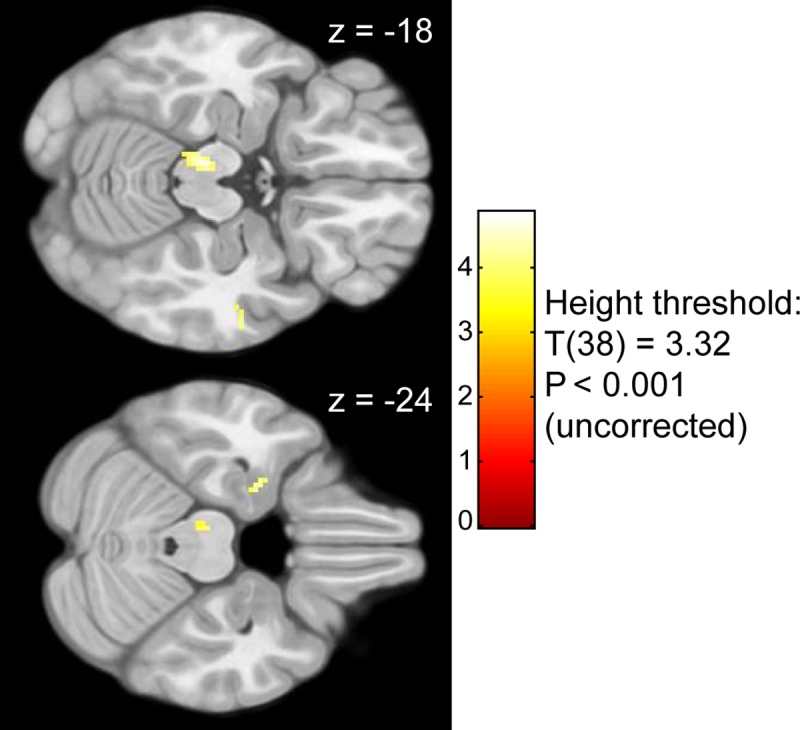
Result for the SPM mass univariate second level contrast [regular products—ONS]. The color-coding of the intensity map is based on T value. The transverse slices indicate higher BOLD responses during tasting regular products than tasting ONS products in the amygdala and temporal pole (z = -24), and ventral tegmental area (z = -18).

To check if we successfully captured BOLD responses associated with flavor processing in the first level analysis and whether there are no large differences between both data sets we carried out a second level mass univariate analysis. Fig 5 and Table 1 shows results on group-level for the main effect of flavor stimulus delivery (P_(FWE)_< 0.05). Cluster size (k) was set to > 100 voxels, to focus on the largest BOLD activation areas. For completeness, we supplied the entire T-map of the contrast (df = 38) in the supplementary materials. As expected, we found clusters of BOLD activation in thalamic, sensory, and motor areas as well as insular regions in response to flavor stimuli. Furthermore, Fig 6 and Table 1 show group differences for the contrast [regular products–ONS] using a more liberal threshold (P_(uncorrected)_< 0.001). The results show that BOLD-responses in the left amygdala, temporal pole and ventral tegmental area were higher in the group tasting regular products. We found no group differences for the contrast [ONS—regular products] using the same threshold. Contrast T-images are available in S1 File and S2 File.

**Table 1:** Peak regions for flavor stimulation across all subjects and between experimental groups.

Contrast^a^	Cluster	Peak Region	Size in voxels^b^	Peak voxel P_(uncorrected)_	Peak voxel P_(FWE)_	T	MNI coordinates		
							x	y	z
Flavor versus Baseline	1	Right Postcentral Gyrus	16788	< 0.001	< 0.001	15.25	60	-8	30
		Right Precentral Gyrus		< 0.001	< 0.001	15.10	66	-2	28
		Right Postcentral Gyrus		< 0.001	< 0.001	13.23	68	-10	12
	2	Left Postcentral Gyrus	9788	< 0.001	< 0.001	13.68	-56	-10	30
		Left Precentral Gyrus		< 0.001	< 0.001	12.56	-62	-12	22
		Left Postcentral Gyrus		< 0.001	< 0.001	12.09	-46	12	-2
	3	Supplementary motor cortex	2466	< 0.001	< 0.001	12.06	0	4	56
		Paracingulate Gyrus		< 0.001	< 0.001	9.60	2	16	42
		Supplementary motor cortex		< 0.001	< 0.005	7.36	8	4	68
	4	Left Dorsolateral Prefrontal Cortex	743	< 0.001	< 0.001	8.52	-34	48	28
		Left Dorsolateral Prefrontal Cortex		< 0.001	< 0.001	8.05	-44	46	18
	5	Septal Area	157	< 0.001	< 0.001	7.83	0	0	6
Regular versus ONS^c^	1	Ventral Tegmental Area	94	< 0.001	0.24	4.50	-8	-26	-18
	2	Left Middle Insula	71	< 0.001	0.45	4.18	-36	-8	22
	3	Middle Temporal Gyrus	31	< 0.001	0.56	4.05	58	-26	-10
	4	Left Amygdala	25	< 0.001	0.58	4.02	-28	-2	-26
		Temporal Pole		< 0.001	0.80	3.74	-34	8	-28
	5	Middle Temporal Gyrus	12	< 0.001	0.70	3.88	54	-10	-20
	6	Left Precentral Gyrus	21	< 0.001	0.81	3.73	-44	4	18

^a^ The contrast [ONS versus Regular] showed no significant results thresholded at p < 0.001, uncorrected for multiple comparisons.

^b^ Voxel size was 2x2x2 mm.

^c^ Statistics are reported thresholded at P_(uncorrected)_< 0.001, and should be interpreted with caution.

#### Second-level ICA

**Fig 7:**
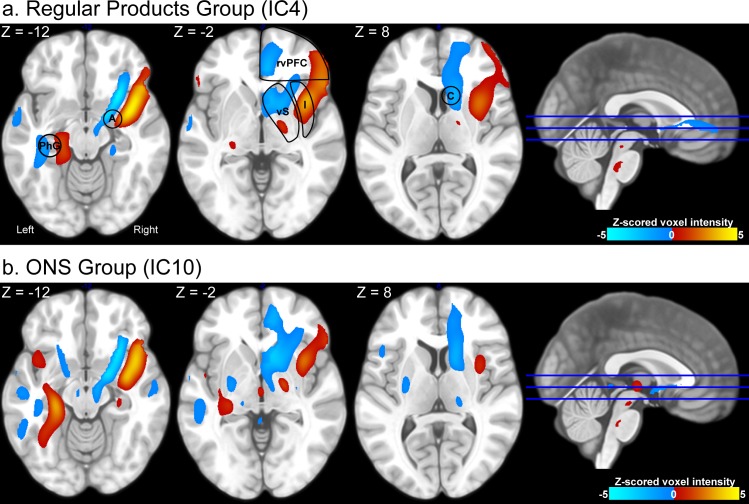
Brain maps encompassing the ventral emotional network. The figure presents spatial maps of independent components that encompass the ventral emotional network in the regular products group (a) and ONS group (b). Images are thresholded at |z| > 1. Brain areas that co-vary (i.e. groups of brain areas similarly colored) within the spatial map are indicated in red (joint increase in BOLD responses) and blue (joint decrease in BOLD responses). Areas indicated in red negatively correlate with areas indicated in blue within the IC. Both components are similar (spatial correlation: Pearson r^2^ = 0.52). Several key areas have been highlighted: the amygdala (A), parahippocampal gyrus (PhG), right ventral prefrontal cortex (rvPFC), insula (Ins), ventral striatum (vS), and caudate nucleus (C). For the regular products group, the depicted IC is significantly associated with flavor pleasantness ratings (see Fig 8).

**Fig 8:**
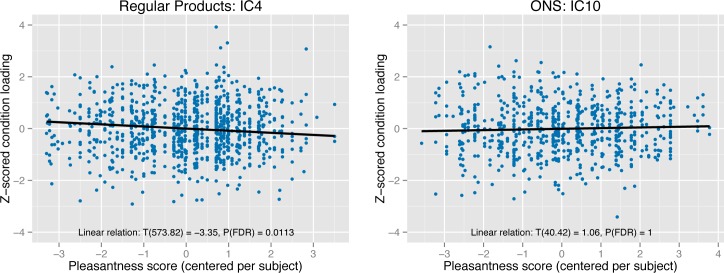
Flavor condition profiles of the ventral emotional network. Independent component analysis (ICA) resulted in component brain maps (see Fig 7) and flavor condition profiles. These profiles refer to independent component loadings per participant per trial and indicate how strong each brain map, depicted in Fig 7, was represented in the brain during each flavor condition. In the current figure, the flavor condition loadings are plotted as Z-scores on the y-axis. As each participant rated flavor pleasantness per trial, we were able to associate the flavor condition loadings with the pleasantness scores. In the current figure, pleasantness scores (mean centered per participant) are plotted on the x-axis. The relation between flavor condition profiles of the current IC and pleasantness scores is shown for both the regular products group (left panel) and ONS group (right panel). The relation was significant in the regular products group (T(573.82) = -3.35, P < 0.001, P(FDR) < 0.05) indicating that a stronger representation of the IC was associated with lower pleasantness scores. Degrees of freedom are computed using the Satterthwaite’s approximation.

In the regular products group, the second level ICA result contained one component that was significantly associated with pleasantness scores (T(573.82) = -3.35, P < 0.001, P(FDR) < 0.05). The spatial map of this component showed large spatial overlap with the ventral emotional network: the component encompassed the left parahippocampal gyrus, right amygdala, insula, ventral striatum and ventral prefrontal cortex. For the ONS group, no component was significantly associated with pleasantness scores. However, we did find one component that was spatially similar to the ventral emotion network component in the regular products group (spatial correlation: r^2^ = 0.52). We found no relational trend between condition loadings of this component and pleasantness scores (T(59.63) = 0.95, P = 0.35, P(FDR) = 1. Spatial maps of the components are given in Fig 7 while Fig 8 indicates the relation between flavor condition profiles of these components and pleasantness scores. An overview of all components is given in S2 Fig. Furthermore, flavor condition profile scores are available in S1 Dataset and S2 Dataset while IC spatial maps are available in S3 File and S4 File.

Note that in Fig 7, areas indicated in red are negatively associated with areas indicated in blue over all stimuli and participants. Furthermore, areas in red are negatively associated with pleasantness scores, while areas in blue are positively associated with pleasantness scores. The results therefore indicate that each depicted area subdivides into a part that is positively associated with pleasantness scores and a part that is negatively associated with pleasantness scores: 1) medial vs. lateral ventral prefrontal cortex, 2) anterior vs. middle insula, 3) anterior vs. more posterior ventral striatum, 4) medial vs. lateral amygdala, and 5) lateral vs. medial parahippocampal gyrus.

## Discussion

To the best of our knowledge, the current fMRI study is the first to investigate flavor pleasantness processing including both tasting and swallowing using a network-based analysis. We aimed to find a hedonic functional brain network associated with pleasantness coding of flavors, and to investigate whether this network encompasses the ventral emotion network defined in [5]. In two separate datasets, we found a functional network showing large overlap with the ventral emotional network. Whereas activity of this ventral emotion network was associated with flavor pleasantness of off-the-shelf drinks, we found no association between this emotion network and pleasantness ratings of oral nutritional supplements. There were no other functional networks within each data set significantly associated with pleasantness scores.

Phillips et al. (2003) [5] associated the ventral emotion network with the amygdala, insula, ventral striatum, and ventral regions of the prefrontal cortex. Although lateralized to the right hemisphere, our analysis included all of these regions within one functional network. Additionally, the network we isolated in the current study encompassed the left parahippocampal gyrus as well. Interestingly, the right amygdala, right insula, right ventral prefrontal cortex and the left parahippocampal gyrus could be subdivided into a part that is positively associated with pleasantness and a part that is negatively associated with pleasantness (see Fig 7).

### Amygdala

Although early neurobiological chemosensory studies associated the amygdala with processing of aversive stimuli only [39–41], other studies indicated that this structure showed activity for pleasant as well as unpleasant chemosensory stimuli [6,42,43]. Moreover, multiple studies have found no correlation between amygdala responses and taste or flavor pleasantness ratings [7,8,44]. Instead, the amygdala was found to be responsive to taste intensity [6,8,45]. Interestingly, these intensity effects in the amygdala were found to interact with stimulus pleasantness. Winston et al. (2005) [9] found that intense odors evoked greater amygdala responses compared to weak odors for an unpleasant and a pleasant stimulus, while there was no difference in amygdala response between intensities of a neutrally pleasant odor. These results indicate that the amygdala is involved in processing the emotional salience of pleasant and unpleasant chemosensory stimuli but not of neutral stimuli. In correspondence with these findings, our study also indicates that the amygdala is associated with both pleasant and unpleasant flavors. Furthermore, co-occurrence of the amygdala, nucleus accumbens and ventral prefrontal regions is in line with connectivity studies of the amygdala indicating (reciprocal) connections between these areas [43,46,47].

Our results indicated that the amygdala showed higher BOLD responses when tasting regular products compared to ONS products. Additional to pleasantness, the amygdala has also been associated with chemosensory stimulus learning [48,49]. Therefore, this result may indicate tasting familiar products involves recruitment of the left amygdala. However, this result should be interpreted with caution as we used a lenient threshold and our experiment was not designed to investigate taste memory.

### Insular cortex

Crouzet et al. (2015) [10] showed that the insular cortex is involved in early taste quality processing. Furthermore, neuroimaging studies indicate that the insular cortex is not only involved in taste or flavor quality processing, but also in processing its associated intensity and pleasantness [6,19,44,45,50,51]. In previous work, we showed that insular processing of these taste characteristics is lateralized; taste quality and taste pleasantness is mainly processed in the left insula, while taste intensity is mainly processed in the right insula [24]. Furthermore, Nitschke et al. (2006) [11] showed that cognitive manipulations altering perceived taste pleasantness (e.g. providing false intensity information on the upcoming taste stimulus), are reflected in insular taste stimulus processing.

In a previous study we found that right anterior insula activity was predominantly associated with taste intensity [24]. Although, we associated the exact same insular area with flavor pleasantness in the current study, these results do not necessarily contradict. Pleasantness and intensity highly correlate. In the current study, we did not manipulate nor measure perceived intensity and, therefore, are unable to disentangle these flavor characteristics.

### Ventral striatum

The ventral striatum, and in particular the nucleus accumbens (NAc), has been associated with processing pleasantness [12,52]. Gottfried et al. (2002) [53] showed the ventral striatum also associates with odor pleasantness learning. These authors paired odor presentations with facial expressions and found that NAc activity increased over time during pleasant olfactory learning. Additionally, ventral striatal activity has been associated with cognitive manipulations in affective value of taste and flavor [54]. Finally, a recent study suggests that the ventral striatum may also be associated with processing aversion [13]. In line with these studies we found that the right ventral striatum positively correlated with the full flavor pleasantness range. Furthermore, ventral striatum activity clustered together with ventral medial prefrontal cortex activity. This is in correspondence with a study by Di Martino et al. (2008) [55] who showed strong functional connectivity between these areas.

### Orbitofrontal cortex / Ventral prefrontal cortex

A growing body of neuroimaging studies associated the orbitofrontal cortex (OFC), (also termed ventral prefrontal cortex in many studies), with pleasantness coding [3,6,7,44,56,57]. The posterior part of the OFC is thought to process multimodal integration of sensory information, while the anterior part processes reward value [3]. Furthermore, an important distinction can be made between the medial (m) and lateral (l) OFC. Whereas the mOFC is associated with reward value processing, the lOFC is associated with the evaluation of punishment, which may lead to behavioral change [3,56,58]. Hayes et al. (2014) [14] performed a meta-study on processing pleasant and unpleasant chemosensory stimuli. The authors found great overlap within OFC areas in response to pleasant and unpleasant stimuli. Differences between pleasant and unpleasant stimuli were found in the mOFC and left lOFC, showing increased activation for pleasant stimuli, whereas unpleasant stimuli were associated with the right caudolateral OFC activity.

In line with the studies mentioned above, we found a clear distinction between the right medial and lateral OFC (or vPFC). We found that the right mOFC was associated with increasing pleasantness, whereas the right lOFC was associated with decreasing pleasantness.

### Parahippocampal gyrus

Interestingly, our analysis also indicated involvement of the left parahippocampal gyrus. Involvement of the hippocampus/parahippocampal gyrus in chemosensory studies has been reported in multiple studies [59–61]. Reviews have indicated that the parahippocampal gyrus may be involved in energy regulation [62,63]. Furthermore, this area has been associated with emotional memory coding and especially when modulated by arousal [64–66]. Future research should elucidate the role of this area in the emotional response to chemosensory perception.

### Limitations

For the ONS stimuli, we were unable to find a relation between flavor pleasantness and the ventral emotion network. This may be caused by multiple limiting factors within this study. First, the experiment was not optimized to reliably evoke pleasantness as well as disgust within each participant. Consequently, the current study suffers from measurement sensitivity with respect to the whole pleasantness range. Second, the unfamiliarity of the ONS products may have led to differences in stimulus evaluation. Although we cannot confirm this hypothesis, as we did not measure familiarity, we found that stimulus evaluation was indeed different as pleasantness ratings in the ONS group decreased during the course of the experiment. Furthermore, we also observed a difference in BOLD signal in the amygdala between both experimental groups, which might be related to chemosensory memory retrieval. This result should be interpreted with caution, however, as the ONS data set included less trials, and therefore less statistical power.

Not finding the relation between pleasantness ratings and the ventral emotion network in the ONS data set may also be related to learning the metabolic impact of a flavor stimulus. In recent work, Araujo et al, 2013 [67], showed that (implicitly) learned metabolic consequences are related to flavor pleasantness processing. It may be that participants did not learn the metabolic consequences of the novel ONS stimuli yet.

## Conclusion

We found that a ventral emotion network (consisting of the ventral prefrontal cortex, ventral striatum, amygdala, insula and parahippocampal gyrus) in the brain is associated with flavor pleasantness perception for regular products. These areas subdivide into a part that is positively associated with pleasantness and a part that is negatively associated with pleasantness. Our results indicate that although a highly similar network can be identified when tasting ONS, pleasantness for these products is processed differently. This could be related to the novelty of these products.

## Supporting information

S1 Dataset Behavioral data and IC loadings ONS data set. The data set contains the Subject ID, fMRI run number, pleasantness scores, product ID, presentation order, mean centered pleasantness score, and the IC loadings. Each data row represents a single trial.
(TXT)

S2 Dataset Behavioral data and IC loadings RP data set. The data set contains the Subject ID, fMRI run number, pleasantness scores, product ID, presentation order, mean centered pleasantness score, and the IC loadings. Each data row represents a single trial.
(TXT)

S1 Fig Joint second-level ICA result: spatial maps and loadings as a function of pleasantness ratings for IC14. The figure presents results of the first independent component from an ICA performed on both datasets together. *Top*: the figure presents a spatial map of the first independent component, which encompasses the ventral emotional network. The image is thresholded at |z| > 1. Brain areas that co-vary (i.e. groups of brain areas similarly colored) within the spatial map are indicated in red (joint increase in BOLD responses) and blue (joint decrease in BOLD responses). Areas indicated in red negatively correlate with areas indicated in blue within the IC. *Bottom*: the flavor condition loadings are plotted as Z-scores on the y-axis against pleasantness scores (mean centered per participant) on the x-axis. As each participant rated flavor pleasantness per trial, we were able to associate the flavor condition loadings with the pleasantness scores. The relation between flavor condition profiles of the first IC and pleasantness scores is shown for both the regular products group (left panel) and ONS group (right panel). The relation was significant in the regular products group (T(933) = -3.09, P < 0.002) indicating that a stronger representation of the IC was associated with lower pleasantness scores. Further, inspection of the results indicated that the relation between this IC and liking was not significantly stronger in the regular products data set compared to the ONS data set (pleasantness x data set interaction: T(1615) = 1.88, p = 0.06). The number of ICs was estimated using the MDL algorithm, which resulted in 16 ICs. Group data was reduced to 24 PCs on an individual level and to 16 PCs on a group level. Degrees of freedom are computed using the Satterthwaite’s approximation. See the main text for the further statistical methods.
(TIF)

S2 Fig IC spatial maps and relation between IC loadings and pleasantness ratings. All spatial IC maps have been ordered based on similarity. The relation between IC loadings and demeaned (i.e. mean centered) pleasantness scores are indicated per IC. T statistics are based on linear mixed effect models (see main text). P-values are corrected for multiple comparisons using an FDR correction.
(PDF)

S1 File Statistical parametric map (NIFTI format) containing group contrast ONS vs. RP. (ZIP)

S2 File Statistical parametric map (NIFTI format) containing group contrast flavor vs. Baseline. (ZIP)

S3 File IC spatial component maps in 4D NIFTI format for the ONS data set. (ZIP)

S4 File IC spatial component maps in 4D NIFTI format for the RP data set. (ZIP)
