# Supplementary figures and images for: Flavor pleasantness processing in the ventral emotion network
Source: PLoS One. 2017 Feb 16;12(2):e0170310. doi: 10.1371/journal.pone.0170310 (PMC5312947; doi:10.1371/journal.pone.0170310)

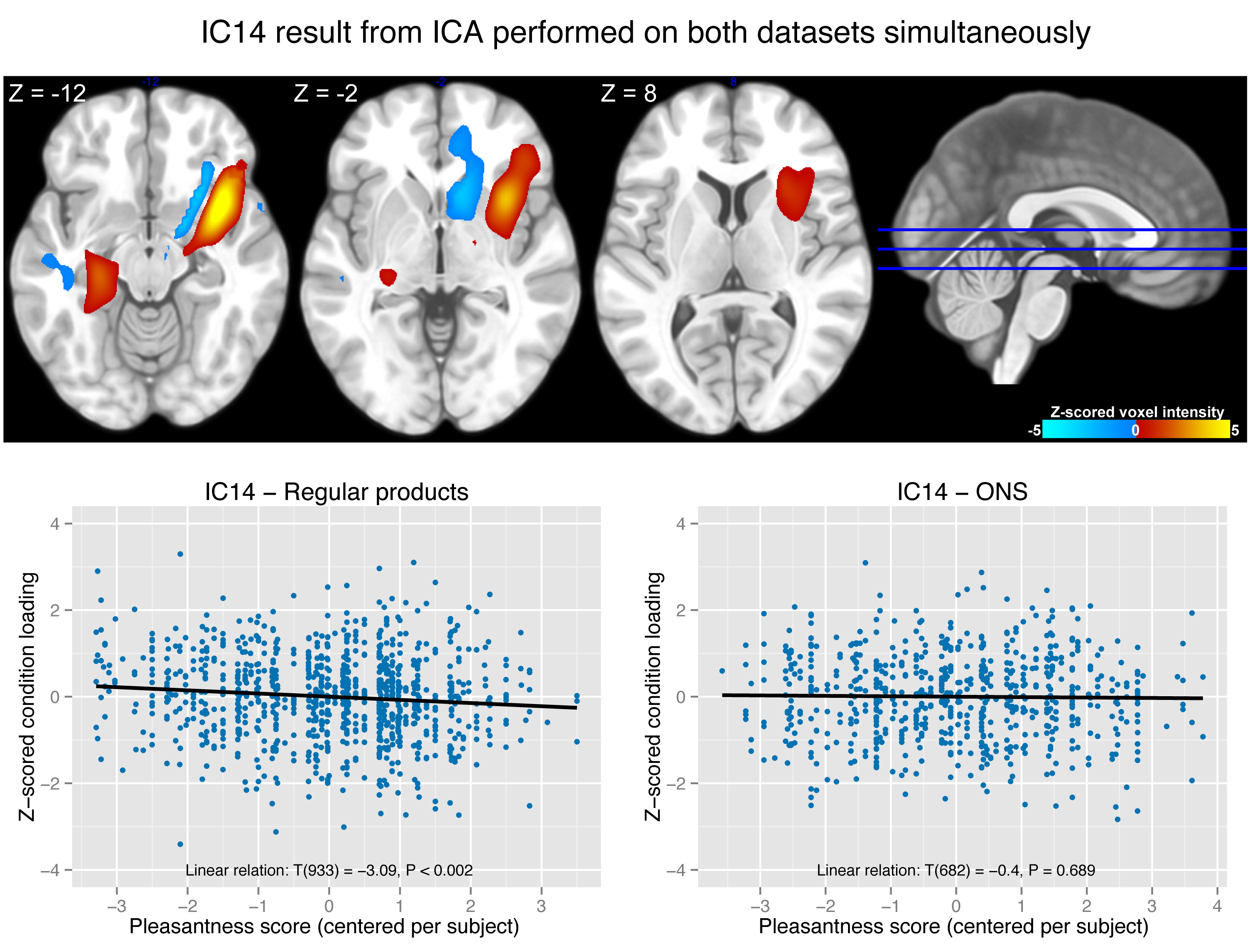

Supplement: S1 Fig — The figure presents results of the first independent component from an ICA performed on both datasets together. Top: the figure presents a spatial map of the first independent component, which encompasses the ventral emotional network. The image is thresholded at |z| > 1. Brain areas that co-vary (i.e. groups of brain areas similarly colored) within the spatial map are indicated in red (joint increase in BOLD responses) and blue (joint decrease in BOLD responses). Areas indicated in red negatively correlate with areas indicated in blue within the IC. Bottom: the flavor condition loadings are plotted as Z-scores on the y-axis against pleasantness scores (mean centered per participant) on the x-axis. As each participant rated flavor pleasantness per trial, we were able to associate the flavor condition loadings with the pleasantness scores. The relation between flavor condition profiles of the first IC and pleasantness scores is shown for both the regular products group (left panel) and ONS group (right panel). The relation was significant in the regular products group (T(933) = -3.09, P < 0.002) indicating that a stronger representation of the IC was associated with lower pleasantness scores. Further, inspection of the results indicated that the relation between this IC and liking was not significantly stronger in the regular products data set compared to the ONS data set (pleasantness x data set interaction: T(1615) = 1.88, p = 0.06). The number of ICs was estimated using the MDL algorithm, which resulted in 16 ICs. Group data was reduced to 24 PCs on an individual level and to 16 PCs on a group level. Degrees of freedom are computed using the Satterthwaite’s approximation. See the main text for the further statistical methods. (TIF) [file pone.0170310.s003.tif]
