# Supplementary material for: Flavor pleasantness processing in the ventral emotion network
Source: PLoS One. 2017 Feb 16;12(2):e0170310. doi: 10.1371/journal.pone.0170310 (PMC5312947; doi:10.1371/journal.pone.0170310)
Supplement: S2 Fig — All spatial IC maps have been ordered based on similarity. The relation between IC loadings and demeaned (i.e. mean centered) pleasantness scores are indicated per IC. T statistics are based on linear mixed effect models (see main text). P-values are corrected for multiple comparisons using an FDR correction. (PDF) [file pone.0170310.s004.pdf]

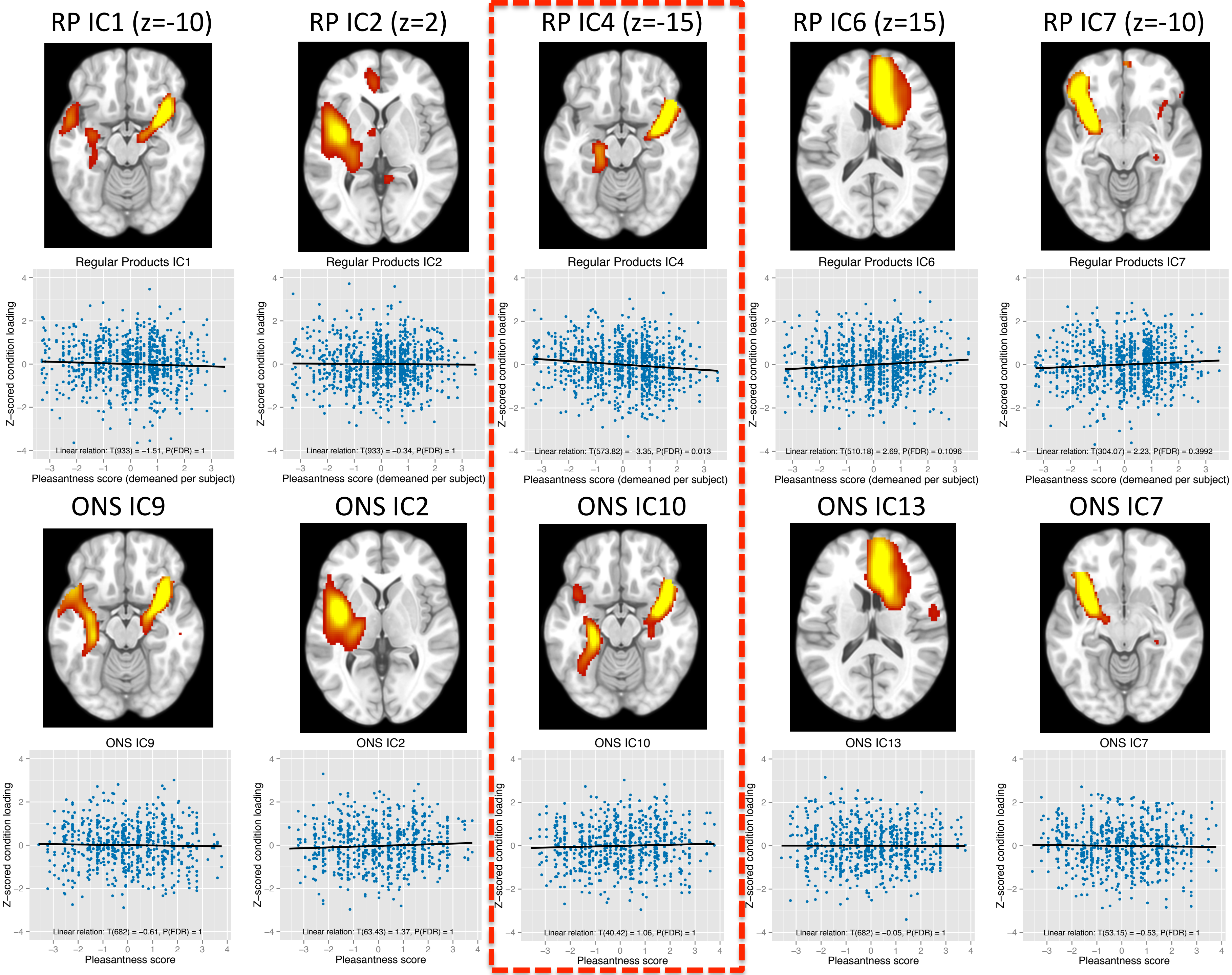

RP IC1 (z=-10)
RP IC2 (z=2)
RP IC4 (z=-15)
RP IC6 (z=15)
RP IC7 (z=-10)
Regular Products IC1
Linear relation: T(933) = −1.51, P(FDR) = 1
Regular Products IC2
Linear relation: T(933) = −0.34, P(FDR) = 1
Regular Products IC4
Linear relation: T(573.82) = −3.35, P(FDR) = 0.013
Regular Products IC6
Linear relation: T(510.18) = 2.69, P(FDR) = 0.1096
Regular Products IC7
Linear relation: T(304.07) = 2.23, P(FDR) = 0.3992
Z−scored condition loading
Pleasantness score (demeaned per subject)
ONS IC9
ONS IC2
ONS IC10
ONS IC13
ONS IC7
ONS IC9
Linear relation: T(682) = −0.61, P(FDR) = 1
ONS IC2
Linear relation: T(63.43) = 1.37, P(FDR) = 1
ONS IC10
Linear relation: T(40.42) = 1.06, P(FDR) = 1
ONS IC13
Linear relation: T(682) = −0.05, P(FDR) = 1
ONS IC7
Linear relation: T(53.15) = −0.53, P(FDR) = 1
Z−scored condition loading
Pleasantness score

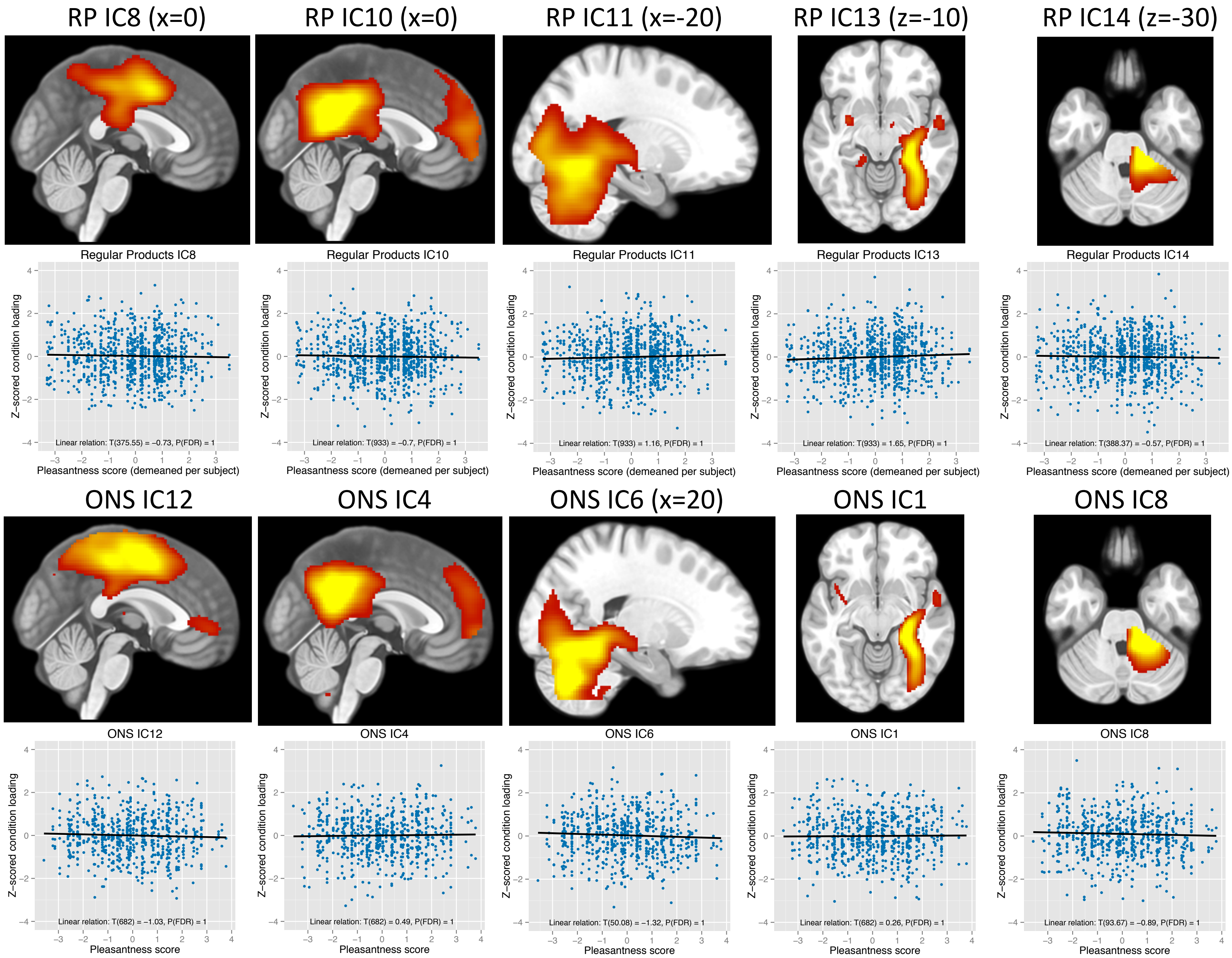

RP IC8 (x=0)
RP IC10 (x=0)
RP IC11 (x=-20)
RP IC13 (z=-10)
RP IC14 (z=-30)
Regular Products IC8
Linear relation: T(375.55) = −0.73, P(FDR) = 1
Regular Products IC10
Linear relation: T(933) = −0.7, P(FDR) = 1
Regular Products IC11
Linear relation: T(933) = 1.16, P(FDR) = 1
Regular Products IC13
Linear relation: T(933) = 1.65, P(FDR) = 1
Regular Products IC14
Linear relation: T(388.37) = −0.57, P(FDR) = 1
Z−scored condition loading
Pleasantness score (demeaned per subject)
ONS IC12
ONS IC4
ONS IC6 (x=20)
ONS IC1
ONS IC8
ONS IC12
Linear relation: T(682) = −1.03, P(FDR) = 1
ONS IC4
Linear relation: T(682) = 0.49, P(FDR) = 1
ONS IC6
Linear relation: T(50.08) = −1.32, P(FDR) = 1
ONS IC1
Linear relation: T(682) = 0.26, P(FDR) = 1
ONS IC8
Linear relation: T(93.67) = −0.89, P(FDR) = 1
Z−scored condition loading
Pleasantness score

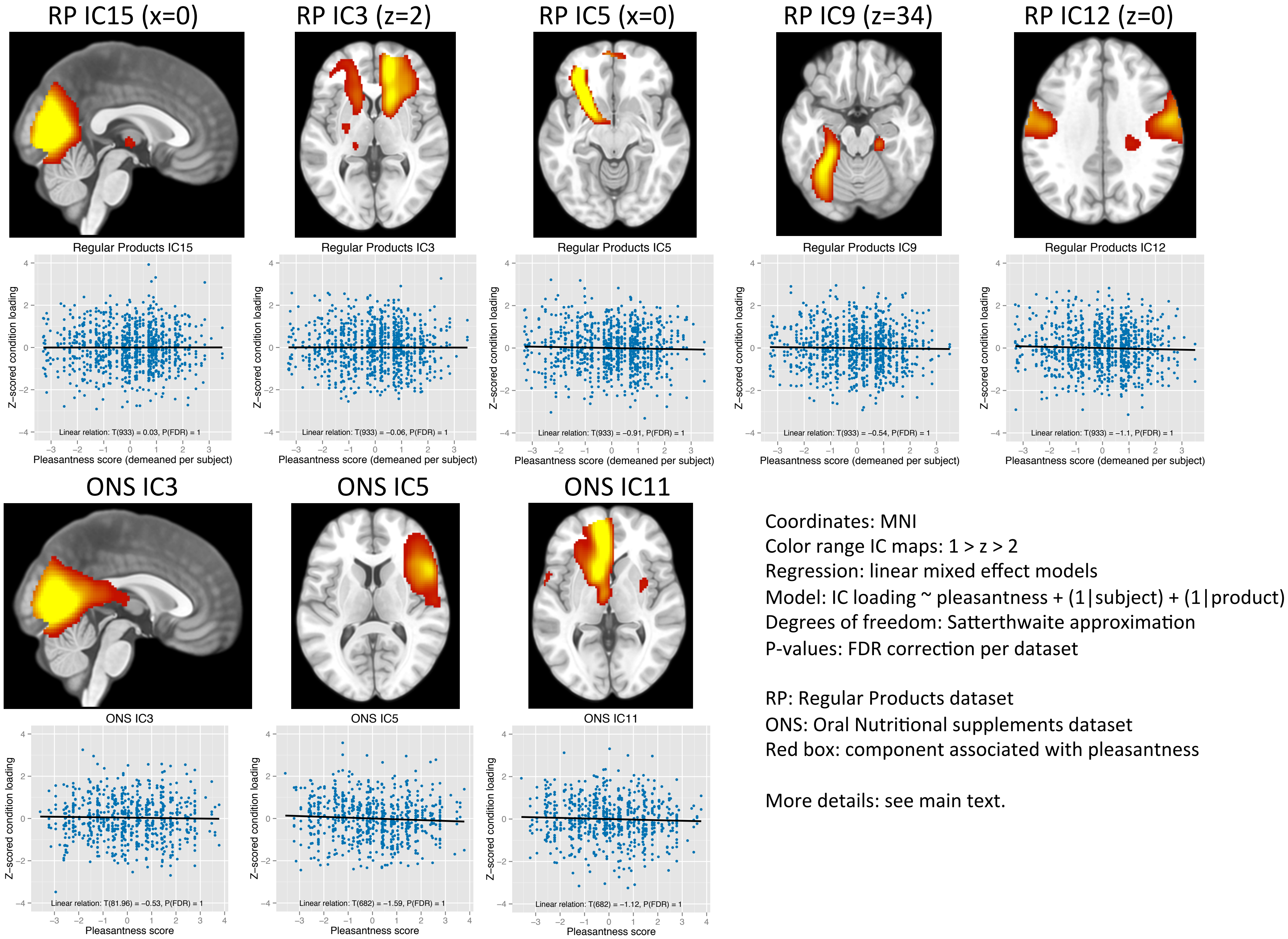
RP IC15 (x=0)
RP IC3 (z=2)
RP IC5 (x=0)
RP IC9 (z=34)
RP IC12 (z=0)
Regular Products IC15
Linear relation: T(933) = 0.03, P(FDR) = 1
Regular Products IC3
Linear relation: T(933) = −0.06, P(FDR) = 1
Regular Products IC5
Linear relation: T(933) = −0.91, P(FDR) = 1
Regular Products IC9
Linear relation: T(933) = −0.54, P(FDR) = 1
Regular Products IC12
Linear relation: T(933) = −1.1, P(FDR) = 1
Z−scored condition loading
Pleasantness score (demeaned per subject)
ONS IC3
ONS IC5
ONS IC11
ONS IC3
Linear relation: T(81.96) = −0.53, P(FDR) = 1
ONS IC5
Linear relation: T(682) = −1.59, P(FDR) = 1
ONS IC11
Linear relation: T(682) = −1.12, P(FDR) = 1
Z−scored condition loading
Pleasantness score
Coordinates: MNI
Color range IC maps: 1 > z > 2
Regression: linear mixed effect models
Model: IC loading ~ pleasantness + (1|subject) + (1|product)
Degrees of freedom: Satterthwaite approximation
P-values: FDR correction per dataset
RP: Regular Products dataset
ONS: Oral Nutritional supplements dataset
Red box: component associated with pleasantness
More details: see main text.
